# Scale and Sampling Effects on Floristic Quality

**DOI:** 10.1371/journal.pone.0160693

**Published:** 2016-08-04

**Authors:** Greg Spyreas

**Affiliations:** Illinois Natural History Survey, Champaign, IL, United States of America; University of Sydney, AUSTRALIA

## Abstract

Floristic Quality Assessment (FQA) is increasingly influential for making land management decisions, for directing conservation policy, and for research. But, the basic ecological properties and limitations of its metrics are ill defined and not well understood–especially those related to sample methods and scale. Nested plot data from a remnant tallgrass prairie sampled annually over a 12-year period, were used to investigate FQA properties associated with species detection rates, species misidentification rates, sample year, and sample grain/area. Plot size had no apparent effect on Mean *C* (an area’s average Floristic Quality level), nor did species detection levels above 65% detection. Simulated species misidentifications only affected Mean *C* values at greater than 10% in large plots, when the replaced species were randomly drawn from the broader county-wide species pool. Finally, FQA values were stable over the 12-year study, meaning that there was no evidence that the metrics exhibit year effects. The FQA metric Mean *C* is demonstrated to be robust to varied sample methodologies related to sample intensity (plot size, species detection rate), as well as sample year. These results will make FQA measures even more appealing for informing land-use decisions, policy, and research for two reasons: 1) The sampling effort needed to generate accurate and consistent site assessments with FQA measures is shown to be far lower than what has previously been assumed, and 2) the stable properties and consistent performance of metrics with respect to sample methods will allow for a remarkable level of comparability of FQA values from different sites and datasets compared to other commonly used ecological metrics.

## Introduction

### FQA

Floristic Quality Assessment (FQA) metrics are used to measure the conservation value of an area via its flora. FQA has continually increased in popularity and its metrics are widely relied upon to directly inform land-use decisions. They are most commonly used to identify valuable natural areas for acquisition, legal protection, and conservation; monitor the progress of restorations [1]; set legal standards for habitat recovery (e.g., compensatory wetland mitigation, [2]); determine the effectiveness of ecological management [3,4]; and answer ecological and conservation research questions [5,6,7,8].

The two primary FQA metrics, the Mean Coefficient of Conservatism (Mean *C*) and the Floristic Quality Index (FQI), are based on the Conservatism values (*C*-values) of the vascular plants in a region (typically a state). Mean *C* is calculated as the average *C-*value of species in a sample area, and FQI is the product of Mean *C* and the square root of species richness. Either an entire habitat patch, or plots (site sub-sample), can be the sample unit. Individual *C*-values in a given state have previously been assigned to each plant species based on their individual likelihood of being found in high-quality, biologically undegraded, remnant natural areas in that state, which is a function of anthropogenic disturbance to habitats [9]. *C-*values range from zero (tolerant of anthropogenic disturbance, no fidelity to remnant habitats) to 10 (intolerant of human stressors, exclusive to remnant habitats). Nonnative species are assigned zeros. The FQA metric values that result from a site’s species’ *C*-values have been shown to accurately measure its anthropogenic disturbance and biological degradation levels [10,11,12]. An area’s Floristic Quality value may therefore be said to measure its conservation value as undegraded habitats have become rare in human dominated landscapes.

The increasing popularity of FQA metrics in part stems from their ease of use and flexibility. The liberal ways that metrics are used and that FQA values are compared occurs despite a relative lack of study regarding the ecological properties of metrics, especially those influenced by sampling methodology. As a result, values from sites with substantial differences in their sampling methods are directly compared with one another. This means that decisions regarding the protection, destruction, and restoration of thousands of hectares of natural areas each year are based on comparing Floristic Quality metrics whose sampling based properties are largely unknown [13]. This understanding gap is in stark contrast to other intensively studied ecological measures such as diversity whose ecological properties are well understood [14]. Given that FQA values directly determine land conservation, policy, and management decisions, their ecological properties must be understood. I seek to assess four critical FQA metric properties: area effects, inter-annual (year) effects, species detectability and species (mis)identification effects.

### Sample Area

Almost all biological metrics respond differently across spatial scales [15]. For FQA calculations, the sample area (plot size, or habitat patch size when an entire site is sampled), often varies across datasets of interest. If FQA metrics are affected by sample area (area effects), comparisons of values from different sites would be of little value. FQI incorporates species richness into its calculation, so its values have a positive relationship with sample area (e.g., [16]). Therefore, studies using FQI values have tended to compare values with the same sized sample areas, or they have controlled for sample area statistically [17,18,19]. Where sample area has not been controlled for, Mean *C* is the preferred FQA metric for comparisons [16,17,18,19]. The critical assumption here is that Mean *C* is not affected by sample area. But, whether or not Mean *C* is an “area-neutral” metric is insufficiently assessed (reviewed in, [13]).

### Species Detection and Misidentification

Species lists will inevitably contain errors due to species misidentifications (a.k.a. misclassification), unidentified species, or overlooked species [20]. It is not known how robust FQA metrics are to these issues; in other words, how many undetected or misidentified species can occur in a sample before FQA metric precision or accuracy declines. With respect to detection, several authors have speculated that overlooked species are not problematic for FQA metrics, and that incomplete species lists may be expected to yield accurate FQA-based analyses [17,21,22]. However, empirical support for this claim is sparse [23]. Large sample efforts may be made to create complete and accurate species lists to counter potential issues of detection and misidentification. However, if generating precise FQA values is only possible with the highest levels of botanical expertise and exhaustive sampling, the utility of FQA is limited [19]. Empirical studies testing the extent to which FQA measures are affected by species misidentifications and omissions are needed, both to assess performance of the metrics, and to assess logistical limitations to their use (e.g., botanical expertise, funding, and time).

### Inter-Annual (Year) Effects

Insufficient understanding of the temporal dynamics of conservation metrics often leads to their misuse [24], and FQA is no exception [25]. FQA values are often compared from sites sampled in different years [5,26]. The assumption that underlies such use is that absent human disturbance, FQA scores remain stable across years [27]− they are not prone to year effects (i.e., intra-annual or seasonal effects as opposed to inter-annual effects on FQA have been previously studied). While this expectation is not tenable for sites undergoing rapid successional turnover whose FQA values are highly labile (as in young restorations, [28]), FQA values in established remnant habitats are expected to be stable. However, some have warned that even in long-established habitats, stochastic fluctuations from natural disturbances (e.g., grazing, fire, flooding, drought), create enough inter-annual variation in species composition that FQA value comparisons across years are ill-advised under any circumstances [29,30]. Even if species composition remains stable, variation in species detectability or identifiability across years due to phenology or any other factors affecting plant apparency could yield differences in measured values. Variation in FQA values across years that is not due to anthropogenic disturbances would lead to erroneous Floristic Quality comparisons if it is excessive, but this is little studied.

This study seeks to answer the following specific questions with regards to FQA consistency, accuracy, and precision:

1. Are FQA measures sensitive to area effects?
2. How sensitive are FQA measures to variation in levels of species detection?
3. How sensitive are FQA measures to variation in levels of species misidentification?
4. Are measured FQA scores in established remnant habitats stable across years (year effects)?

## Methods

**Fig 1:**
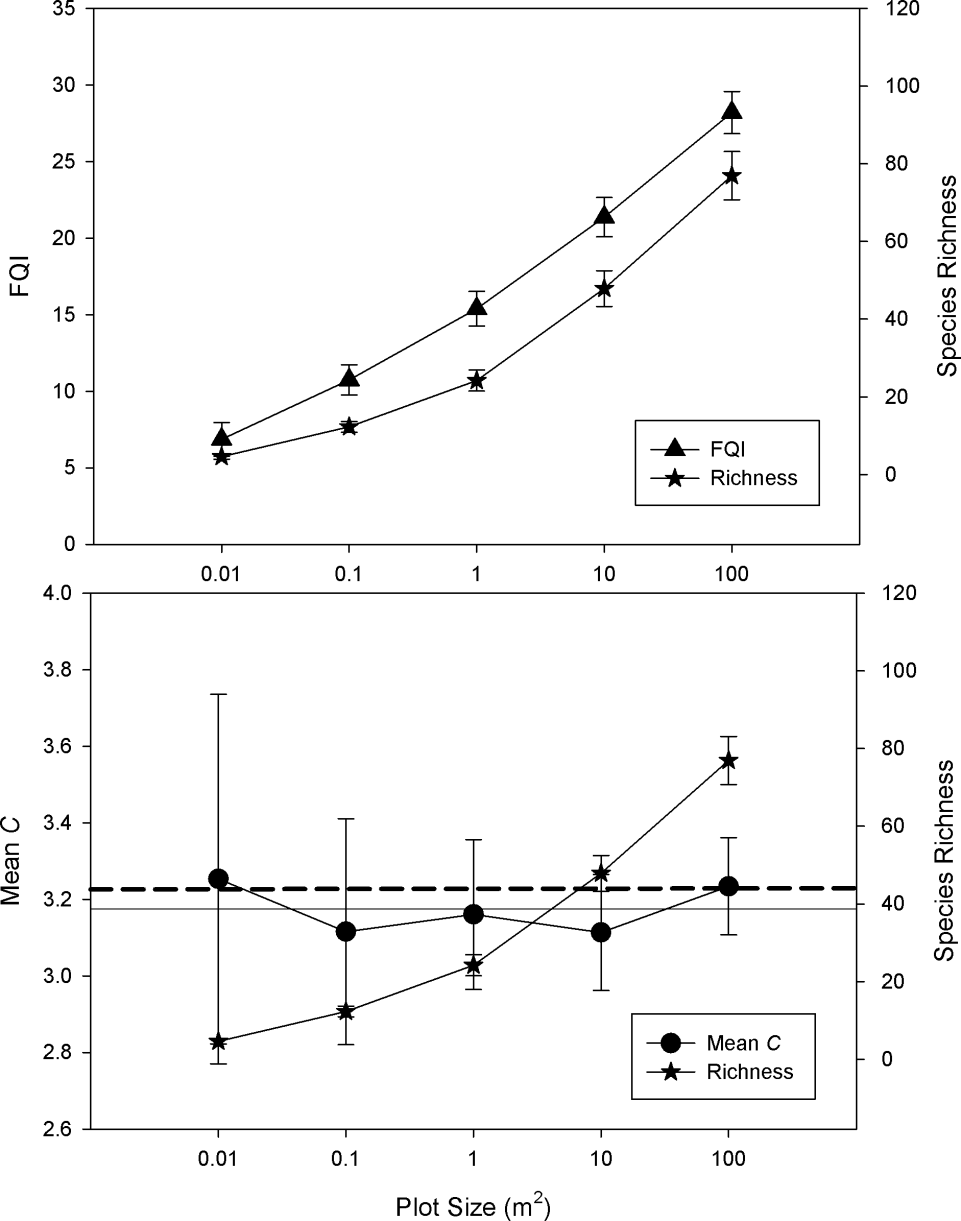
Average FQA and species richness across plot sizes (±95% CI; N = 20). Horizontal lines provide reference levels for comparison. Solid horizontal line indicates average of Mean *C* points (0.01…100), dashed horizontal line is the 100-m^2^ plot value.

This study used vegetation data from a 15,410 ha remnant (unplowed) prairie on the Tallgrass Prairie Preserve in Osage County, north-central Oklahoma, USA (TPP) (located between 36.73° and 36.90° N latitude, and 96.32° and 96.49°) [31]. Twenty randomly placed, permanent plots were sampled annually from 1998–2009 (plot layout illustrated in Fig 1 in, [32]). Plots were randomly placed, except that areas with greater than 20% woody cover, standing water, or exposed rock were avoided. Presence of all vascular plant species were recorded in nested square plots: 0.01, 0.1, 1.0, 10, and 100-m^2^. A single botanist identified species in June each year, thereby avoiding multi-observer inter-observer biases or error [33].

The goal of this study was to assess FQA metric properties from a habitat that had little human disturbance, and was relatively homogenous in human disturbances and ecological gradients. TPP met these criteria as it is over a decade removed from historic human disturbances, which were relatively minor. Plots were placed across the same vegetation type. In 1993 ecological management was initiated with bison (*Bos bison*) grazing and prescribed fire rotations (for further site description and sampling methods see, [31,32]).

Outside of hydrophytic species, species Coefficients of Conservatism (*C*-values) had not yet been assigned to the majority of Oklahoma’s flora (http://www.oklahomaplantdatabase.org). Therefore, *C*-values were used as the mean of the two states nearest the site, Missouri and Kansas [34,35], and the available Oklahoma values. Nearby averaging has previously been used where values are absent (e.g., [17]). Two species from the dataset, *Euphorbia tetraspora* and *Rubus ostryifolius*, were excluded from analyses because they did not have *C*-values from reference states. They were assumed to be erroneous identifications [36]. Any records that could not be identified to species were excluded from analyses, this was less than 1% of the records.

For spatial scale, species detection, and species misidentification analyses, only data from the most recent sample year (2009) were used. For nested plots (10–0.01m^2^), vegetation data from one of the four corners was used. For detectability and misidentification analyses only 1 and 100 m^2^ were used because they are considered to be the most commonly used vegetation study plot sizes. To generate increasingly lower species detection levels, the rarest species (number of occurrences across all plots) were incrementally dropped from the analyses (at 5% increments) based on the assumption that rarer species are more likely to be missed.

For species misidentification analyses, I made the assumption that species were most likely to be mistaken for other species within their functional group. The functional groups deemed most likely to be misidentified were, from most likely to least: annual sedges, perennial sedges, annual grasses, perennial grasses, annual forbs, perennial forbs, ferns, biennial forbs, shrubs, vines, trees. These rankings were based on 1) surveys of expert botanists, and 2) published literature suggesting the most commonly misidentified species groups [19,20]. Therefore, for any given analysis, between 0% and 50% of the species *C*-values were randomly replaced with *C*-values from within the same functional group. Only the annual sedge through annual forb group categories were needed to reach 50% misidentification.

The replacement misidentification species were chosen from two potential pools. The entire TPP species list the across all years, and the county-wide species pool (generated from, http://www.oklahomaplantdatabase.org). Therefore, the first case assumed that a species was being mistaken for another species from the site (157 km^2^) within the same functional group, and the second assumed that it was being mistaken for another species within the same functional group from anywhere across the county (5,970 km^2^).

## Results

Comparisons across different plot sizes show that FQI and richness values increased in parallel across scales (Fig 1). Mean *C* values on the other hand show no trend across scales, although variation in Mean *C* values was largest at the smallest scales (Fig 1; S1 Fig).

**Fig 2:**
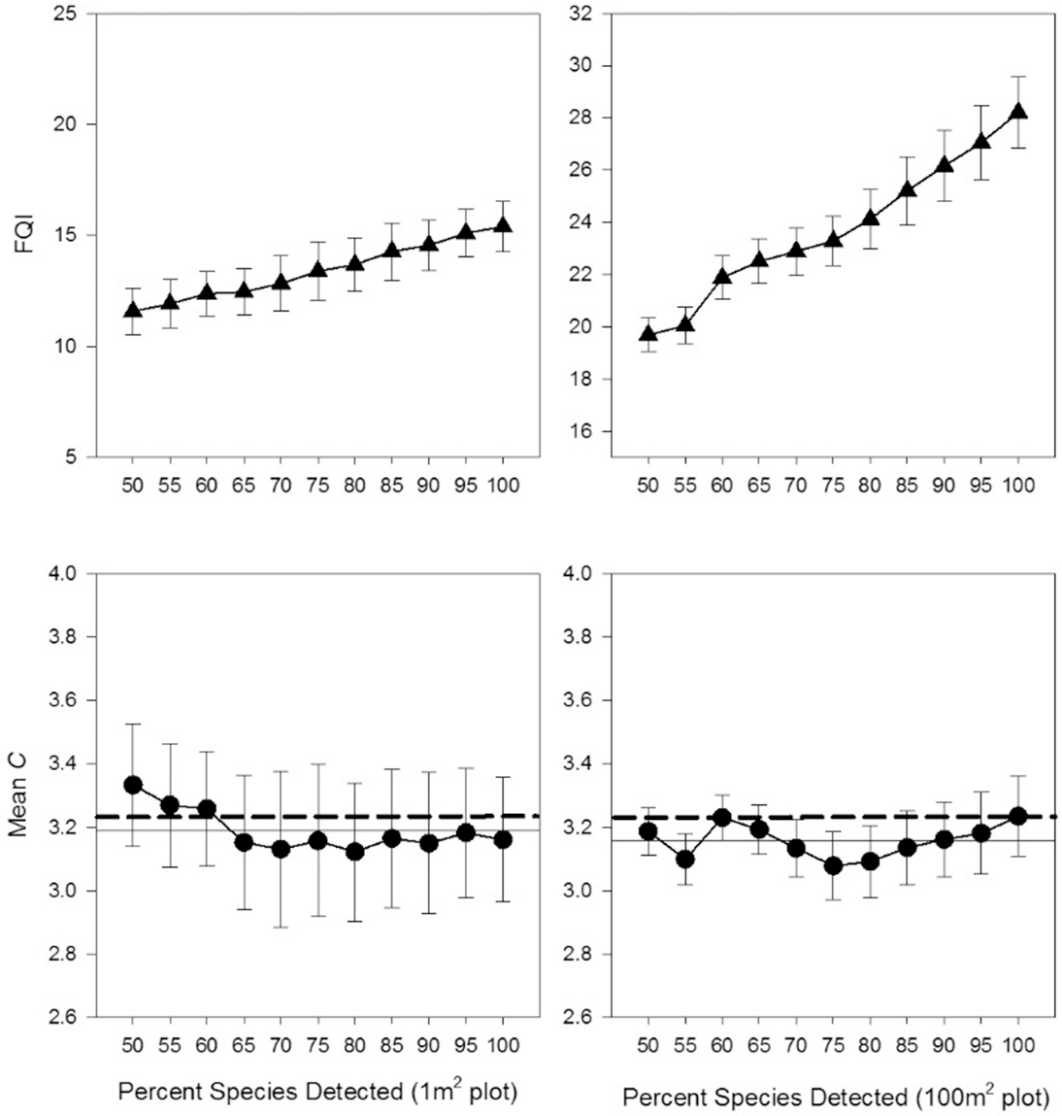
Average FQA values based on 100%–50% of a plot’s potential species detected (±95% CI; N = 20). Horizontal lines provide reference levels for comparison. Solid horizontal line indicates average of Mean *C* points for that plot’s size (50…100%), and dashed horizontal line is 100% detection value in the 100-m^2^ plot.

For species detectability in 100-m^2^ plots, there was no consistent trend in Mean *C* values across detection levels, nor were there large changes in the variation of values as fewer species were detected (Fig 2). For 1-m^2^ plots, there may have been a trend towards slightly higher Mean *C* values at the lower detection level, although their confidence intervals still overlapped (Fig 2). Not surprisingly, in both plot sizes, FQI increased as more species were detected (due to increasing richness).

**Fig 3:**
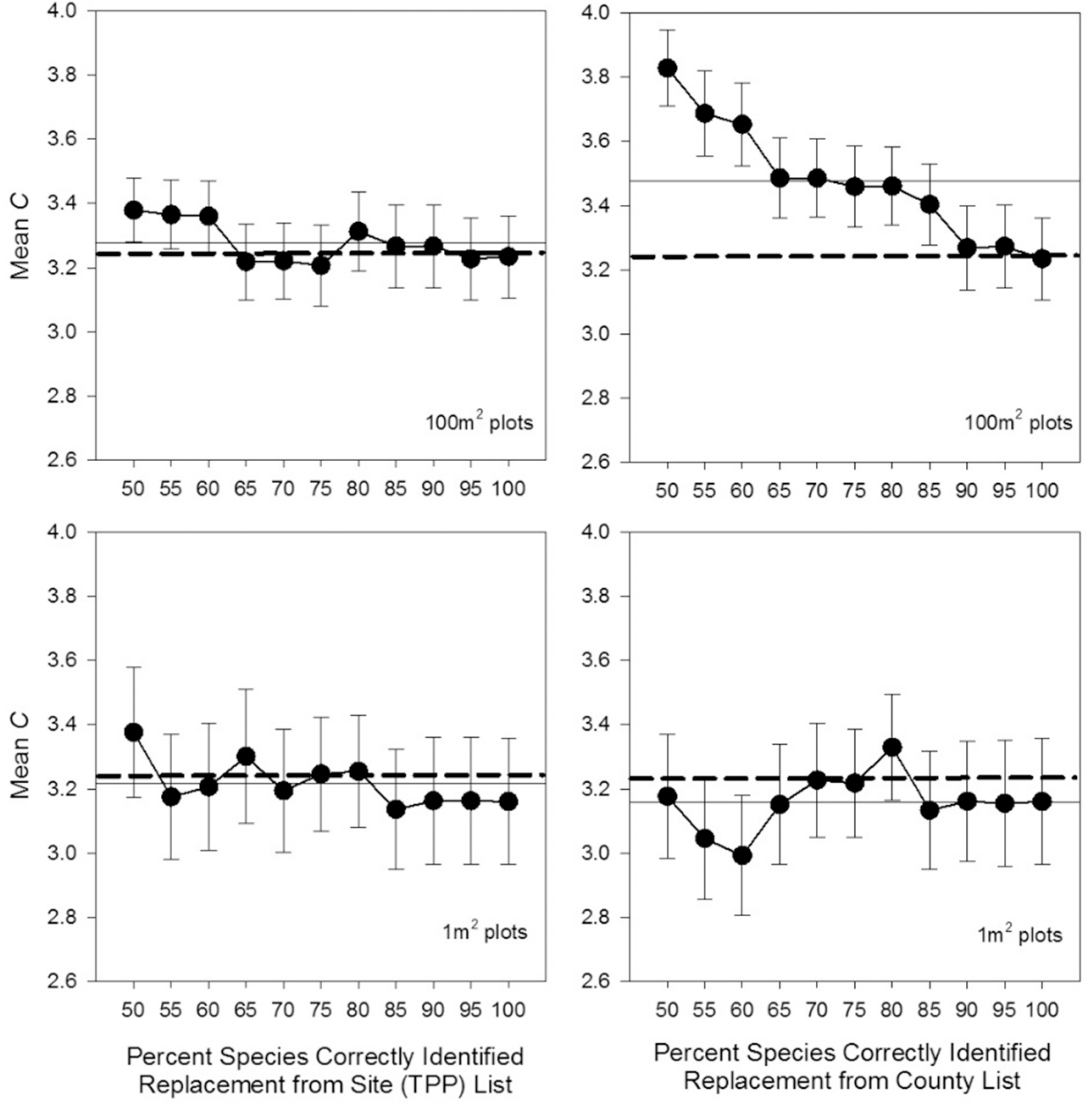
Misidentification Mean *C* values (±95% CI; N = 20). Species replacement values were obtained from the overall site species list (left), and from the county species list (right). Horizontal lines provide reference levels for comparison. Solid horizontal line indicates average of Mean *C* points for that plot’s size (50…100%), and dashed horizontal line is 100% correct identification value in the 100-m^2^ plot.

Outside of a slight increase in Mean *C* at the highest misidentification levels, species misidentification had little apparent effect on Mean *C* values of either plot size when species were misidentified from the pool of other species on site (from the same functional group) (Fig 3). However, when species replacement values were from the larger county species list pool, Mean *C* values in the 100-m^2^ plots quickly increased above a 10% misidentification level (Fig 3), although there was no corresponding trend in the 1-m^2^ plots (Fig 3).

**Fig 4:**
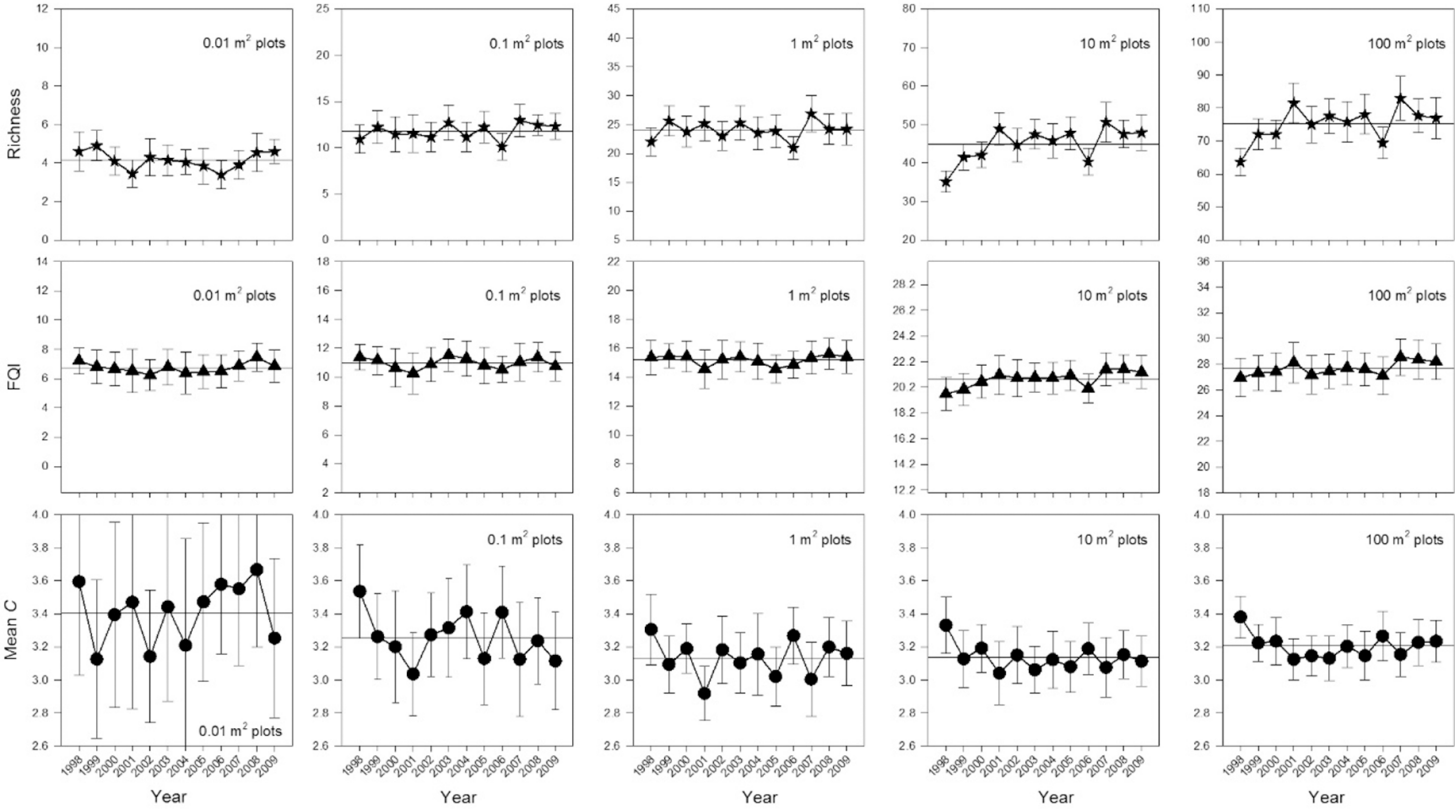
FQA values across sample years for five different plot sizes (±95% CI; N = 20). Horizontal lines provide reference levels for comparison. Horizontal reference line indicates average of points for that plot’s size (1998–2009).

With respect to inter-annual trends, species richness in the largest plots was lower in the earliest sampling years and stable for the remainder of the surveys, while richness in the smaller plots showed no inter-annual trends (Fig 4). FQI and Mean *C* were generally stable over time (Fig 4) with two exceptions. Mean *C* had slightly higher values in the first year’s samples, and once again it had increasingly more variation in values as plot size decreased.

## Discussion

These results demonstrate that Mean *C* does not appear to be subject to area effects across the sample scales examined. On the other hand, FQI is dictated by richness and the species-area relationship. Mean *C* values did show considerable variation around the mean at the smallest scales sampled (< 1 m^2^). However, these smallest plot sizes were well below typical vegetation sampling units and are not relevant for FQA’s typical use. Species detection results showed that low detection Mean *C* values (as low as 60% 1-m^2^ plots and 50% 100-m^2^ plots), were nearly equivalent to complete samples. Furthermore, variability in values did not increase as detection levels declined, meaning that the stability of the metric was maintained even as fewer plants were observed. Species misidentification levels of greater than 60% correct identification (100-m^2^ plots) did not affect Mean *C* when species were mistaken for other plants across the site. When species were replaced (misidentified) from the entire county’s species pool, Mean *C* values became inflated at less than 90% correct identification levels (100-m^2^ plots). No trend was found in 1-m^2^ plots, although its values were inconsistent below 65% correct identification. Measured FQA values across years were relatively consistent, other than aberrant values in the first year’s sample. This suggests that FQA measures are not subject to year effects.

### Area effects

Vegetation researchers of all types grapple with the appropriate sample area, extent, and intensity needed to achieve adequate site sampling [37], and species-area relationships often underlie these considerations. The current study reaffirms previous work that shows that the FQI-area relationship is dictated by species richness (Fig 1), so readers are directed to the considerable body of literature on the species richness-area relationship for FQI’s area based properties [14].

The relationship between Mean *C* and area is unstudied to this point. Authors often anecdotally suggest that a relatively small portion of a site needs to be inventoried to produce a robust site Mean *C* measure, but no specific guidelines have been provided [17,22]. Rooney and Rogers [19] suggested that adding sample plots to a site left its Mean *C* value relatively unaffected. But, to truly isolate the “area” relationship, nested sample plots are needed, because adding more sample plots increases the spatial extent of the sample as well as the area. Area (plot size, sample grain) and spatial extent have very different ecological properties [38]. Results from the present study provide strong evidence that the Mean *C* metric is not subject to area effects. It sets a specific range of plot sizes across which Mean *C* can be confidently assumed to be area-neutral (1–100 m^2^). This “area-neutral assumption” will allow for considerable freedom for FQA users to compare Mean *C* values across studies whose sampling varies in spatial scale.

**Fig 5:**
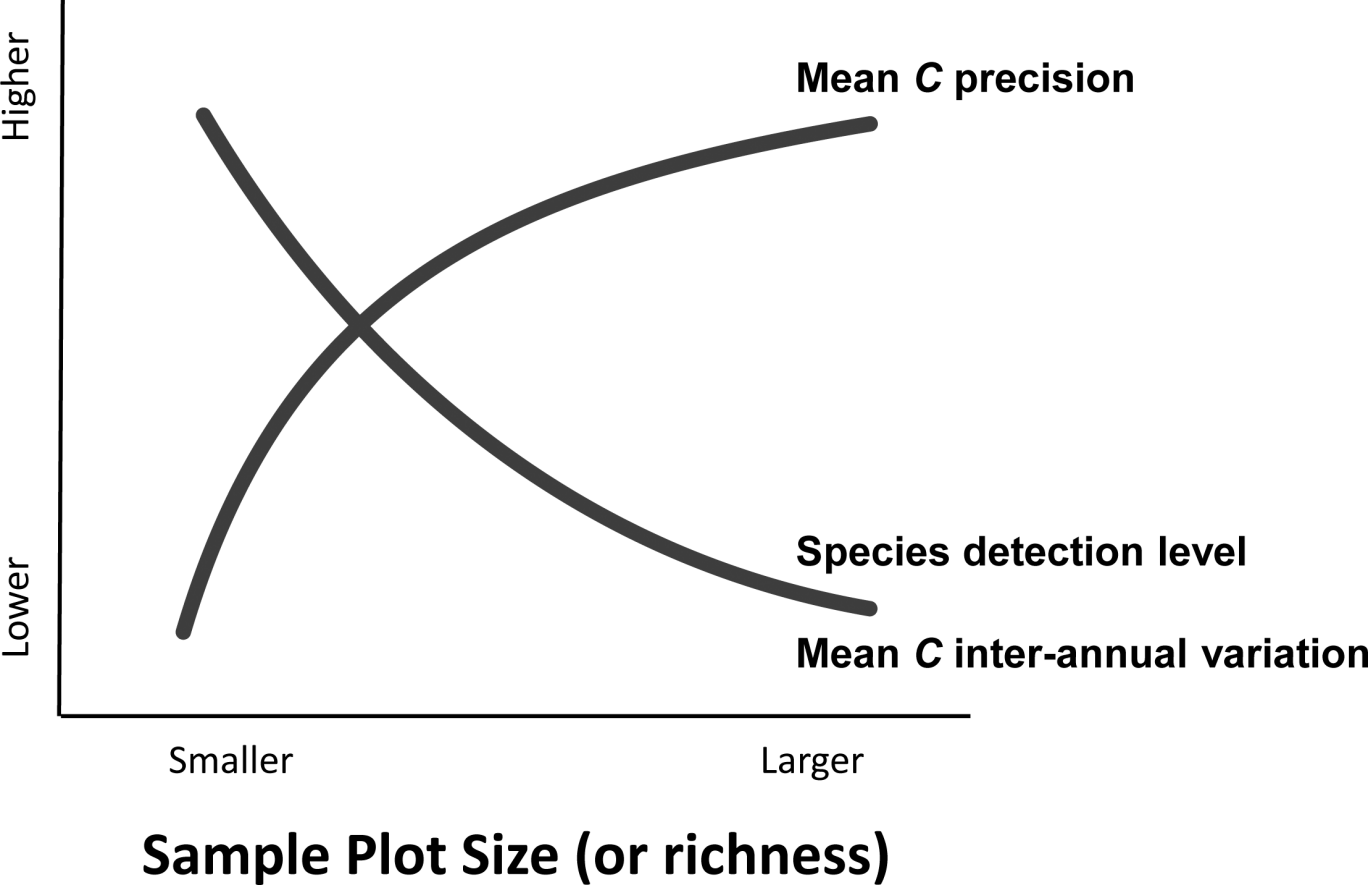
Generalized relationship between common sampling/analysis considerations and Mean *C*. Plot richness can be important to consider when sampling/comparing habitats with different diversity levels.

While it is not realistic to use 20, 0.01-m^2^ plots to sample the vegetation of an area the size of the TPP, analyzing such small plots was a useful exercise for elucidating the properties of FQA measures, as the precision of Mean *C* was lower in smaller plots (S1 Fig). This phenomenon can be attributed to two causes: first, as sample plots become smaller, compositional differences (and β-diversity differences) between them inherently increases [38,39]. Second, fewer values contributing to a mathematical mean will tend to inflate variation, so when few species are present in a sample plot Mean *C* is prone to volatility. In this study, 4.6 species on average were found in the smallest plots (0.01-m^2^), compared to 72.9 in the largest (100-m^2^). Therefore, Mean *C* users should be wary of sample areas with low richness, even if sample plots are of adequate size. Young habitats, highly invaded areas, or inherently low species density habitats could therefore be vulnerable to lowered precision in the Mean *C* metric (Fig 5). For example, Spyreas et al. [26] suggested that by using small plots when sampling low diversity plant communities dominated by an invasive exotic species, their Mean *C* estimates had highly inflated variances. Conversely, it is interesting to note that FQI’s variance did not vary across plot sizes (Fig 1). This suggests that FQI calculated at the plot/quadrat level may be a useful substitute Floristic Quality metric for comparing areas with very small (or species poor) sample plots.

### Species Detection

Incomplete ‘species detectability’ in wildlife studies is widely recognized, and it is typically accounted for statistically [40]. While the causes and consequences of missed plant species in vegetation samples are increasingly studied, vegetation studies have yet to adopt similar controls for the inevitability of species being overlooked [33,41]. With respect to generating accurate Mean *C* values, wide ranges in the acceptable levels of species detection have anecdotally been offered (e.g., between 40%-80% of a site's species,[42]). The current study’s results places the necessary minimum species detection levels at surprisingly low levels (≈ 60% of species in sample plots). Not only do these results show that decreased species detection does not lead to systematic changes to Mean *C* values, but it also does not add variation to values. These findings are especially surprising given that site vegetation inventories during the growing season will typically yield a plant species list that is only about 80% complete when based on a single site visit, due to phenology or other factors that affect species apparency [43]. Therefore, when one considers that site sub-sampling with plots only yields a fraction of the species that would be found in an entire site inventory (note the lack of a richness asymptote in Fig 1), and that only 80% of *that* possible fraction can be found with a single sample, the species detection level in the present study was a far smaller fraction of the site’s overall flora than the percentage value would indicate. Nevertheless, Mean *C* remained consistent at or below even 60% *of that small fraction*.

Some studies have looked at the effects of calculating FQA metrics based on select sub-sets of species in a site’s flora, which is functionally similar to this study’s detection test. Plant roups have been excluded from FQA metric calculations to facilitate more rapid sampling (e.g., only record common or abundant species, [10]), or scores have been calculated using specific sub-set of floras to answer targeted ecological questions (e.g., forbs only, graminoids only, [27]). Results from these sub-set FQA calculations have not been conclusive. For example, removing graminoids from calculations had no effect on Mean *C* in one study [23], while removing Carex species underestimated Mean *C* in another [19]. In general, the current study suggests that FQA score generation based on sub-sets of plant assemblages has a good likelihood of generating accurate Mean *C* estimates because full species lists and their *C*-values appear provide a high level of redundancy in information about the Floristic Quality of an area. This is further demonstrated by the strong correlations among co-occurring species’ *C*-values that occurs within plant communities [9]. The utility of using specific sub-sets will probably depend upon the relative Conservatism level of the specific species groups being omitted/chosen for calculations, which can be very different from the overall flora’s average (S2 Fig).

### Misidentification

Species misidentification rates are considerable in nearly every instance where they have been tested for [44]. While overlooking species is typically more frequent than misidentifying species in vegetation studies, effects from misidentifications are less well understood [20]. The effects of misidentifications on FQA have not been studied to this point. They might be especially consequential for FQA because they directly introduce erroneous integers (*C*-values) into calculations (rather than simply omitting values as in species detection).

When species in sample plots were replaced with values from the TPP species list there was no detectable change to Mean *C*. This analysis approximated a common scenario, where plot sampling was conducted with the aid of an existing site species list for reference, so that botanists would tend to (mis)record species already known from the site. In this case, the lack of evidence for an effect on Mean *C* values despite very high levels of species misidentification might have been expected because of our assumption that the site was relatively homogenous in its human disturbance levels. Thus, if species are replaced with species from a habitat of similar Floristic Quality and human disturbance levels, a similar Mean *C* value is expected.

However, when species were replaced with values from all habitats across the county, estimated values quickly become inaccurate (>10% misidentification 100-m^2^ plots). As misidentification rates increased, Mean *C* values increased from 3.24 at 100% identification (100-m^2^ plots), towards the Mean *C* calculated from the entire county species list (3.68; S2 Fig). This may highlight the outsized influence of introducing rarer, high *C*-value species into the plot species list when using a randomized species replacement procedure. In general, high *C*-value species occur relatively infrequently, and they are therefore less often sampled in plots. But, high *C*-value species make up a relatively high proportion of values in many statewide species lists (9 and 10 are often two of the largest categories, [45]). Thus, under the randomized species replacement protocol, more high *C*-value species would have been added to the plot species list than would normally be found co-occurring in vegetative plots, leading to higher Mean *C* values.

Therefore, it is not only the percent of mistaken species that are important, but also the type of species. Certain groups are more difficult to identify, and certain groups are also more Conservative on average (S2 Fig). It is not known, however, if botanists tend to misidentify more Conservative species for less, or vice versa. If there are patterns to misidentifications this could lead to directional biases in FQA scores. Less skilled botanists might be more likely to mistake lower *C*-value species for higher value ones when using dichotomous keys, because of the relatively large proportion of high *C*-value species in statewide floras. Alternatively, Bernthal [46] suggested that effects from both misidentifications and undetected species are likely to be qualitatively different depending on the skill level of the botanist: “More skilled observers are likely to identify more species and therefore generate higher FQI values. More skilled observers are also likely to find the more Conservative species [high *C*-value], and would tend to generate higher Mean *C* values.” Although less skilled botanists frequently report fewer species in vegetation samples overall, studies have found conflicting results as to whether they more often misidentify rarer species for more common ones, or vice versa [47,48,49,50].

Therefore, it is not clear how instructive the 10% incorrect identification threshold found in the present study is for FQA’s typical use, because randomized misidentifications (within functional groups) might not be realistic for most vegetation sampling. In this study common species such as *Ambrosia artemisiifolia* and *Poa pratensis* were replaced with high *C*-values (0 with 8, and 0 with 5, respectively)–which may represent an unrealistic scenario. Furthermore, random replacement introduced species that might never be found in certain habitat types (e.g., forest species in grasslands, hydric species in uplands). Even an inexperienced botanist could quickly eliminate mismatches of this type by consulting a published flora. Finally, even in habitats with extremely high species richness, where plant identifications are challenging, misidentification rates rarely exceed 10% [33,50]. In total, this suggests that the current study’s results may overestimate the importance of effects from misidentification.

In conclusion, given the current dearth of plant taxonomic and field identification skills [49], it is not surprising that authors would propose that the botanical expertise needed to accurately identify large numbers of plants to the species level is the single largest drawback and logistical limitation to using FQA. However, results in the present study suggest that Mean *C* can tolerate a considerable number of undetected or misidentified species in a sample before its accuracy declines. This should assuage concerns that the time and expertise needed to generate accurate FQA measures is excessively burdensome or restrictive for most FQA uses.

### Year effects

Regular patterns in Floristic Quality over long-term, successional, time scales are beginning to emerge. Floristic Quality in early-successional habitats is always low, and it is usually highly labile and unpredictable from year-to-year (e.g., [28]). As communities enter mid-successional stages, year-to-year volatility dampens and values reach a predictable plateau of modest Floristic Quality [25]. Finally, FQA values in established habitats that have not been degraded, or are long removed from human disturbance, are higher and they are presumed stable. As opposed to directional trends associated with succession, the present study focused on whether an established and presumably stable remnant habitat exhibited significant inter-annual fluctuations in its measured Floristic Quality.

The results support the conclusion that FQA measures in remnant prairie were largely free of year effects (especially in larger sample plots). Differences across years were relatively minor and values were never far from the overall mean, and they appeared largely un-patterned. The exception was the first year of the study, which showed lower richness and higher Mean *C* levels. This was likely an artifact of naïve exposure to the species in the plots by botanists during the first round of sampling [51]. Previous study on inter-annual fluctuations in FQA scores has not produced consistent results. Studies have found site scores sampled in two different years to be highly correlated (e.g., [52]). While this demonstrates high levels of temporal correlation, it gives FQA users no indication of how different actual site scores might be across years (S3 Fig). The only positive finding of year effects in FQA metrics thus far has come from a study of North American prairie pothole wetlands. Euliss and Mushet [29] found that these wetlands underwent dramatic variation in species composition and FQA values, when precipitation and ground water levels varied across years. However, this study used a somewhat unorthodox sample method. It compared values from specific hydrologic zones (concentric rings around the wetland) across years, which probably led to less representative results than if the whole wetland community had been sampled [53].

It is important to remember that inter-annual variation could originate from two proximate causes: 1) changes to species detection/identification among years, or 2) actual changes to species composition. Species detection/identification errors (a.k.a. pseudo-turnover) can be quite high among years for any number of reasons–phenology, weather, sampling effort, botanist expertise, observer error, etc. [54]. Alternatively, fluctuations in actual species composition are possible, for example, with a rapid appearance of annual species after a disturbance. The weak influence from detection and misidentification on Mean *C* values in this study suggests that actual changes in species composition would be the more important of these possible causes (e.g., real extinctions or colonizations). To this point, several authors have warned that short-term species composition changes due to natural disturbances could make FQA measurements unstable or useable [30,55]. Or, at a minimum, Mack et al. [11] and Wilson and Bayley [56] recommend not sampling a community if an extreme weather event such as drought or flooding has occurred because such samples might not be representative of the site over the long-term. However, in addition to considerable annual variation in temperature and precipitation, the TPP grasslands underwent rotational grazing and prescribed fire over the course of the study period that was designed to mimic natural disturbance regimes. But, even these did not appear to yield observable effects beyond the minor inter-annual variation in FQA values that was observed–suggesting a stable measure over time.

Disturbance, or changes in disturbance regimes, lead to exotic species establishment and invasion [57], which can depress FQA values [26]. A question of interest is whether habitats that are prone to regular or cyclic large scale disturbances (river floodplains, coastal wetlands, or tidal zones), would exhibit observable fluctuations in FQA scores across years, or whether they would be more buffered from such changes than other habitats because their floras were assembled with these regular disturbance events. Conversely, are discrete and irregular disturbances, such as extreme drought, hurricanes, wind storms, ice damage, herbivory-grazing, or saltwater intrusions more likely to lead to acute effects on scores (e.g., from exotic species invasions) in less-regularly affected habitats? Long-term studies of natural disturbances and plants species composition are needed to better understand their potential inter-annual effects on FQA metrics. Because management treatments often seek to replicate natural disturbances (fire, grazing, etc.), insights into these questions about the effects from natural disturbance events on FQA could come from studies of ecological management that have associated plant species composition data. For example, a 5-yr woodland fire reintroduction study did not find dramatic inter-annual variation in FQA values, beyond initiating a long-term increase in Floristic Quality [58].

### Conclusions and Future Study

Taken as a whole, this study suggests that FQA metrics, and Mean *C* specifically, exhibit properties that allow for consistent and robust Floristic Quality estimates with varying plot size, species detectability, species misidentification, or sample year. Therefore, it suggests that comparing Mean *C* values from sites or datasets that differ with respect to these factors is possible. The results also show the overall sample effort needed to generate meaningful Floristic Quality measurements to be far below what has previously been assumed.

Similar studies in other vegetation types and regions are needed to confirm the generality of these results. Because different states have assigned *C*-values to their floras in different ways, similar studies in other states are especially important. Investigations of different habitat types with lower or higher diversity and human degradation levels would also be invaluable. While the relationship between species detection and FQA seems remarkably robust, studies assessing botanist ability, time of day, inter-annual phenology, or sample extent would further define the real-world sampling limits to FQA. When choosing a sampling design, this study shows several benefits to using larger sized plots for FQA (Fig 5). This is because even though species detection levels are known to be lower in larger plots, detection levels are shown as relatively unimportant for Mean C, while Mean C precision increases with plot size. However, larger plots take considerably more time and effort to sample, so the tradeoff between using fewer large and more small plots must be balanced. Future study should also assess whether undetected or misidentified species in real-world samples are random or patterned, and if they are patterned, whether they tend to produce directional shifts and bias in Mean *C* values. Finally, opportunistic mining of historic vegetation datasets or ecological management studies would be useful to address questions regarding the effects of large natural disturbance events on FQA measures across years.

## Supporting Information

S1 Fig Standard deviation in Mean *C* values across plot sizes (N = 20). (DOCX)

S2 Fig Average *C* values of the species within the functional groups used in the county species replacement (based on county species list). The overall county list Mean *C* and the Mean *C* of all species found in the TPP plots in 2009 is shown for comparison.
(DOCX)

S3 Fig Variation in Mean *C* values across sample years (N = 20) for 1-m^2^ (top) and 100-m^2^ plots (bottom). (DOCX)
