# Supplementary figures and images for: Scale and Sampling Effects on Floristic Quality
Source: PLoS One. 2016 Aug 4;11(8):e0160693. doi: 10.1371/journal.pone.0160693 (PMC4973955; doi:10.1371/journal.pone.0160693)

**S1 Fig.**
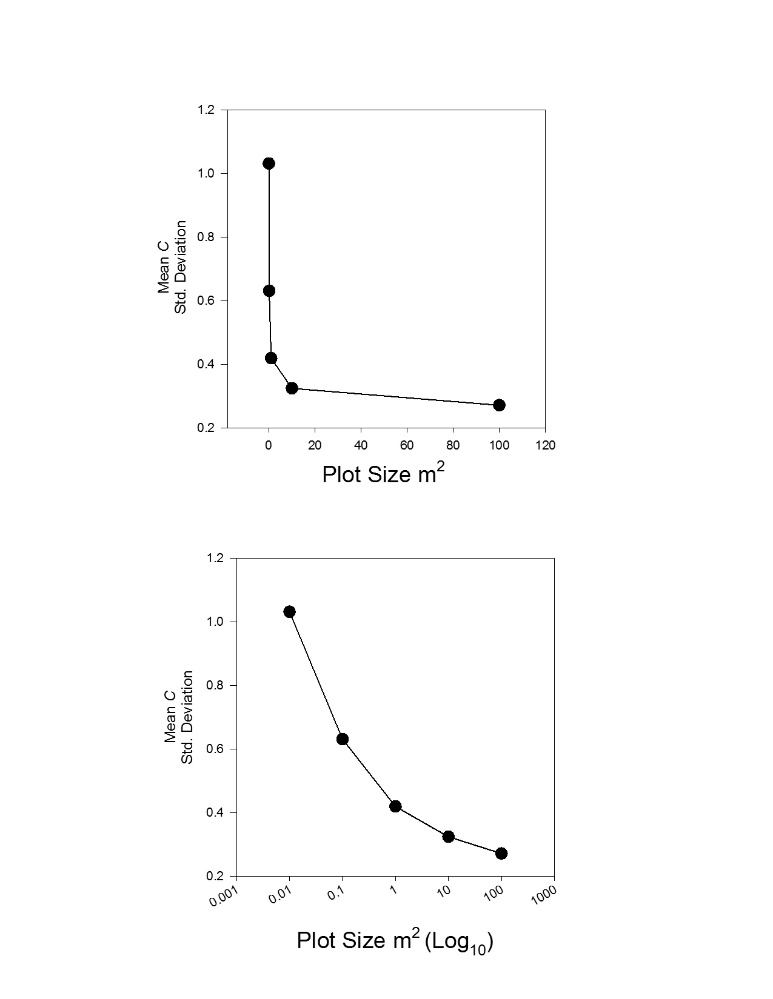

Supplement: S1 Fig — (DOCX) [file pone.0160693.s001.docx]

**S2 Fig.**
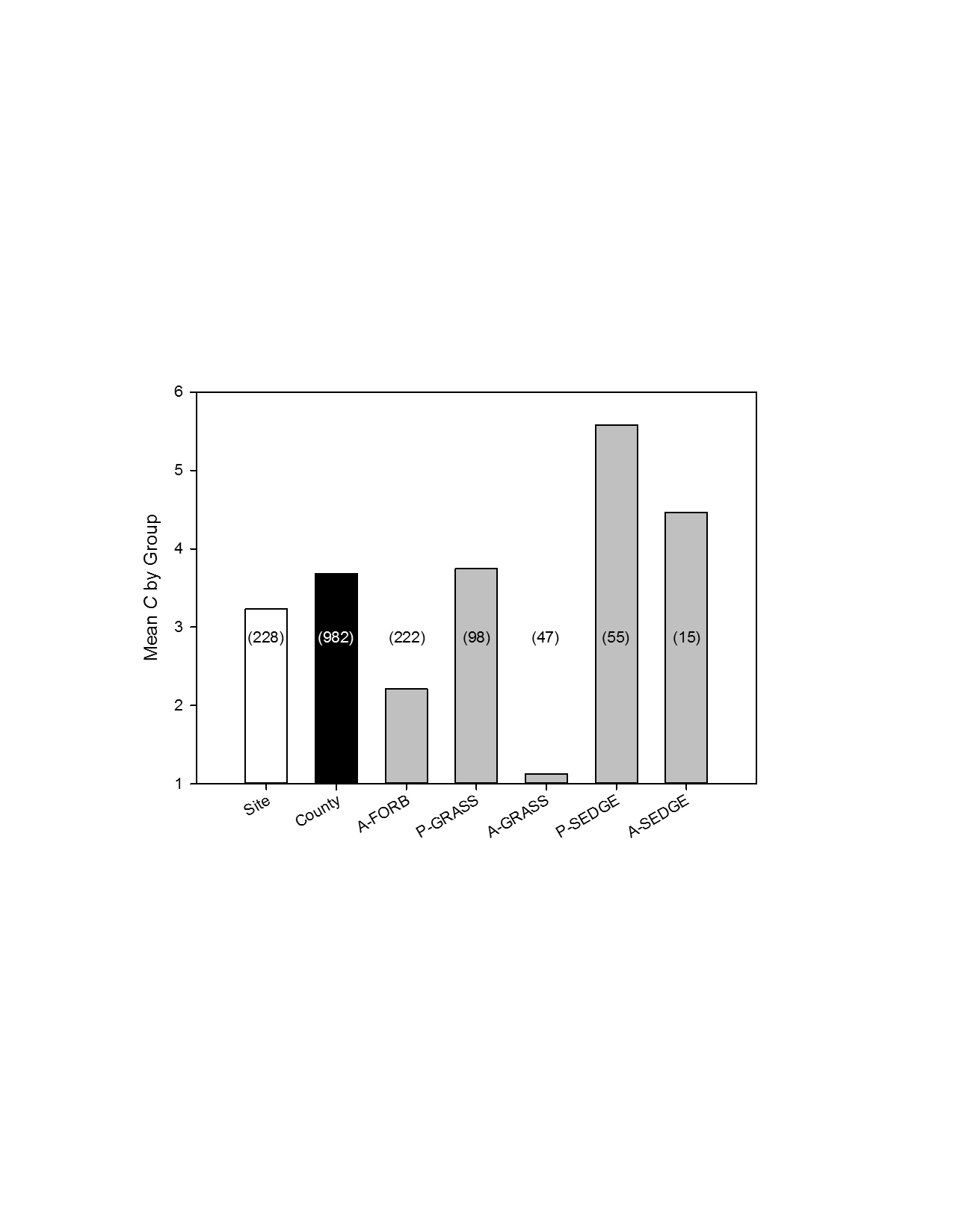

Supplement: S2 Fig — The overall county list Mean C and the Mean C of all species found in the TPP plots in 2009 is shown for comparison. (DOCX) [file pone.0160693.s002.docx]

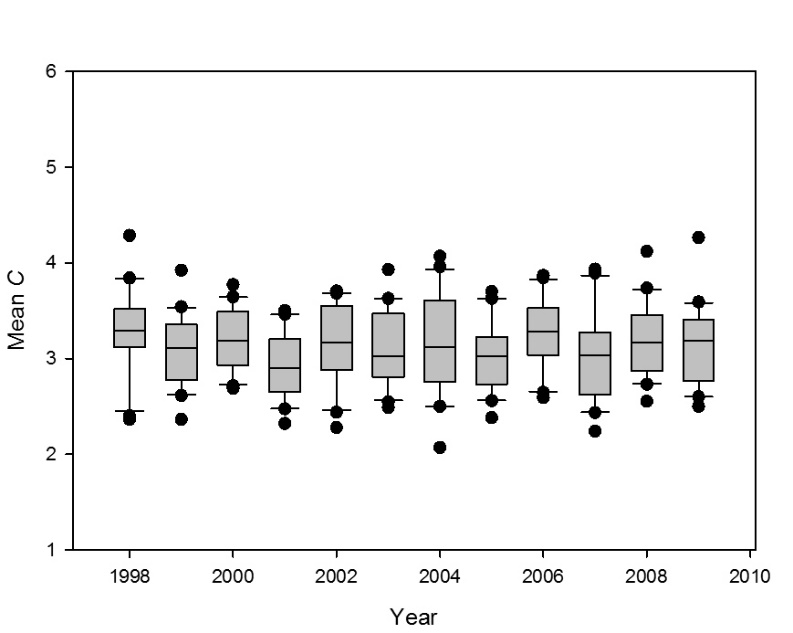


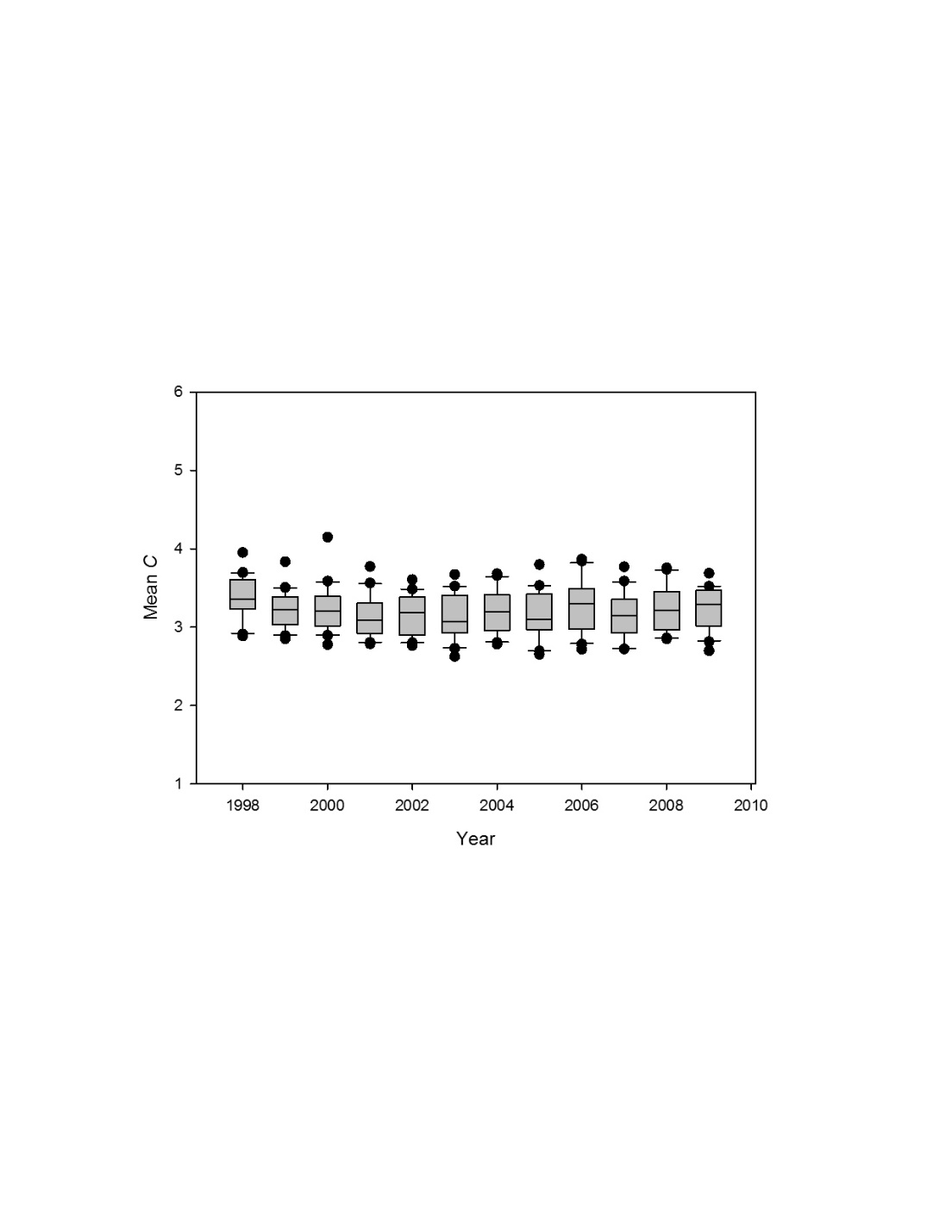


**S3 Fig.**

Supplement: S3 Fig — (DOCX) [file pone.0160693.s003.docx]
